# Parental Self-Efficacy As a Determining Factor in Healthy Mother-Child Interaction: A Pilot Study in Iran

**Published:** 2014

**Authors:** Zohreh Abarashi, Karineh Tahmassian, Mohammad Ali Mazaheri, Leili Panaghi, Nader Mansoori

**Affiliations:** 1 Center of Psychological Counseling Services, Ferdowsi University, Mashhad, Iran.; 2 Family Research Institute, Shahid Beheshti University, Tehran, Iran.

**Keywords:** Child, Interaction, Mental Health, Parental Self-Efficacy

## Abstract

**Objective:** Parental self-efficacy is associated with increasing mental health in children. There is a paucity of research in Iran on the role of parental self-efficacy in increasing mental health status of children. In this preliminary study, we studied the effectiveness of the World Health Organization international child development program (2002) for children aged 3 years and younger in increasing positive interaction between child and mother as an important component of mental health.

**Methods:** Forty mothers from a central hospital child-caring center in Mashhad participated in the study and were randomly divided into an experimental group (n = 20) and a control group (n = 20). The experimental group received the World Health Organization international child development program for 6 consecutive weeks while the control group remained without manipulation. The Parenting Self-Agency Measure (PSAM) was administered at pre-test, post-test and at 1-month follow-up.

**Results: **The study findings indicated that parental efficacy in the experimental group increased after 6 consecutive weeks of training and remained unchanged after 1 month of follow-up while the control group showed no difference regarding scores obtained at three stages of the study.

**Conclusion:** International programs such as the World Health Organization international child development program can increase positive child-mother interaction as an important factor which contributes to mental health in early years of life but further studies with larger samples in longer terms and with more follow-up periods in Iran are suggested.

**Declaration of interest:** None.

**Clinical Trial Registration-URL: **http://www.irct.ir. Unique identifier: IRCT2012073010445N1.

## Introduction

Promoting mental health status of young children through increasing parental self-efficacy is a significantly important issue in the field of studies related to the mental health of young children. In first years of life, children are developed in the social and emotional capacities so as to prepare them to experience confidence, to be intellectually inquisitive and capable of relating well to others (1) which is sometimes called early childhood mental health and can increase capacity to experience, manage emotions, and develop close relationships with others such as mother. 

Studies suggest that the first-three years of children’ lives are the bases of development in subsequent developmental life stages and any disruption in the development of this period could dramatically influence other stages of lifespan (2). However, one study showed that 7% of children aged 3-4 years have serious behavioral problems (3), and a further study showed that between 15-21% of children exhibited emotional and behavioral problems (4). In addition to have a high prevalence, emotional and behavioral problems in children predict an increased risk of a range of health and psychiatric disorders including depression, anxiety and conduct behavior (5-9). 

Parent-child interaction has long been considered as a pathway into the relationship and a forecaster of the child’s developmental competence (10) which correlates with the child’s positive cognitive development (11). Inability of the mother to interact in appropriate ways can negatively influence the child’s future cognitive and physical development (12). 

Parental behavior during child’s first five years of life is critical for the development of important social and cognitive outcomes in children and their mental health that establish life-long adaptation and healthy functioning. The interactions and experiences children have in family interaction provide a framework for how the child will interpret interactions and give meaning to events. Studies in developed countries show that parental self-efficacy is important in child mental health. Positive proactive parenting is associated with high self-esteem and social competence, and can be protective against later disruptive behavior and substance misuse (13, 14). 

It has been argued that promotion of the mental health in children is critical to the prevention of mental disorder throughout the lifespan (15). This may also indicate the role for early intervention programs designed to improve parent-child interaction in particular, and parenting practices more generally.

Studies in developed countries show that parenting programs which are aimed at the parents of children, have the potential to inhibit the occurrence of many mental health problems and psychiatric disorders in them. Recent reviews have demonstrated that these parenting programs can achieve positive outcomes for parents and children (16, 17).

To provide experiences that facilitate growth and development of mental health, parents must understand their children's’ needs (18). Early educational interventions are designed, in a large part, to promote healthy parent-child interaction (19) which is one of these ways. With respect to parent-child interaction outcomes, early intervention programs have reported mixed results. Perceived self-efficacy also has been associated with wellbeing among mothers (20). A better understanding of parents’ perceived parenting self-efficacy and associated beliefs, thoughts, and feelings about parenting a child with autism may lead to more supportive interventions that enhance parental well-being.

Some researchers argue that promotion of the mental health in young children is the key component to the prevention of mental disorders throughout the lifespan (15). This may also emphasize a role for early interventions designed to improve positive parent-child interaction in particular, and parenting practices such as the World Health Organization (WHO) international child development program for promoting the psychosocial development of child by improving the interaction between child and his/her main caregiver. 

The WHO’s international child development program is aimed at improving the mother-child interaction so that their infants may be able to obtain a good condition of mental health. In this program, the caregivers go through a process of becoming more conscious of their own skills at providing loving care and guidance which enables her to be effective without being dependent on the trainer, training manuals or on special equipment. This international program does not impose new ideas on caregivers, but promotes the best of local practices of child care (21, 22). 

Best to authors’ knowledge, no study in Iran has ever investigated the effectiveness of the World Health Organization international child development program in improving interaction between children and their caregivers and increasing parental efficacy on mothers of 3 years old and younger children. To contribute to this understanding, the current study was designed to assess the effectiveness of the World Health Organization international child development program on parental efficacy.

## Materials and Methods

***Participants***

The study had an experimental design and the sample was recruited through poster emonstration in one of the main and central child-caring centers. A sample of 40 mothers with average socio-economic status who were main caregivers of their children voluntarily participated in the study and were randomly divided in to two experimental group (n = 20) and control group (n = 20).

The two groups were matched based on the ages of children, education of mothers and socio-economic status of the families. The majority of children in both groups were female. Mean (±standard deviation, SD) age of children in the experimental group was 16.5 (±8.3) months and in control group it was 18.2 (±7.3) months. Mean (±SD) age of mothers in experimental group was 29.1 (±3.9) years and it was 28.7 (±3.9) years in control group. The majorities of mothers in both groups had 14 years of education and less and were typically employed (Table 1).

Inclusive criteria for mothers included having children of three years of age or younger, being the main caregivers of their children, no current use of drug and alcohol and no prior participation in the World Health Organization international child development program training or similar parenting programs. Participation was voluntary and confidential. All the mothers were given written consent forms and the protocol of the study was approved by the institutional review board of Shahid Beheshti University in Tehran, Iran.

***Questionnaires***

*Demographic checklist*

A demographic checklist designed by the researchers was applied to collect demographic characteristics of mothers and their children such as gender, age, education and socio-economic status. We selected those mothers who were in middle socio-economic status according to their monthly family earning (average income of 1,100,000 Iranian Rials).

*Parental Self Agency Measure*

Dumka et al. devised the Parental Self-Agency Measure (PSAM) to evaluate efficacy of mothers in behaving with their children (18). This scale includes 10 questions with 5 positive questions and 5 negative questions. This scale measures general parental self-efficacy. The PSAM questions are ranged on a 7-item Likert scale ranging from 1 = seldom to 7 = always. In the study of Tylor, the validity and reliability of the PSAM via test-retest was 54% Cronbach’s alpha (23). In the study of Talei, the reliability of the Persian version of PSAM was 70% Cronbach’s alpha (24). In this study, the reliability and validity of the PSAM were 71% via test-rest within two weeks which were satisfactory.

***Study Procedure***

All the study procedures were conducted weekly in a quiet room designated to the study in Razavi child-caring center. Demographics and the PSAM were completed first and then the mothers were randomly divided into two experimental and control groups. The experimental group participated in 6 one-hour weekly sessions practicing the World Health Organization guidelines for good interaction between mother and child. Each session was followed by a home task based on the last modified version of the World Health Organization program for improving mother and child interaction while the control group received no education.

In the 1^st^ session, the psychologist initiated the session with introducing the program and discussion on appropriate interaction between the mother and child by delivering the booklet "*Eight Guidelines for Good Interaction*" to mothers, containing the guidelines and initiated the first guideline with practicing showing positive feelings to the child. This booklet was designed in Persian and was obtained from the WHO program. In the 2^nd^ session, the psychologist initiated the session with reviewing the previous meeting and explained emotional expressive communication of appropriate interaction and the mothers were presented with the guideline two consisting of talking to child and getting a conversation by presenting emotional expressions, gestures and sounds and continued with role playing. The 3^rd^ session was initiated with group sharing and practicing the guidelines 3 and 4 including following the child leads (wishes and body language) and giving affirmation for what the child managed to do well and helping for focusing attention. In the 4^th^ session, group discussion continued and mothers were presented with the concept of mediated learning experience and guidelines 5 consisting of helping the child to focus his attention and share his/her experiences. The 5^th^ session was initiated with group discussion and continued with presenting the guideline 6 consisting of helping the child to make sense of his/her world by sharing and describing it. In the 6^th^ session, the psychologist initiated this session with reviewing of the previous meetings and then continued with group discussion sharing of personal examples of interaction related to guidelines 7 and 8 consisting of helping the child to expand and enrich his/her experiences and learn rules, limits and values. In this session, all the guidelines were reviewed and the group was encouraged to freely talk about what they had learnt and how they thought the training program had helped them to improve their interactions with the children. This session ended with summing up the main points highlighted in the discussions in this session and reiterating the aims of the caregiver/child interaction for better psychosocial development of the child. The mothers were also encouraged by the psychologist to put into practice all the concepts learnt during the program and expressed confidence in the mothers’ ability to do so.

The experimental and control groups completed the PSAM after the end of the last session again (post-test stage) and at 1 month follow-up stage.

***Data Analysis***

The data were analyzed by performing the Chi-square test, independent t-test and variance analysis with repeated measuring in SPSS for Windows 16.0 (SPSS Inc., Chicago, IL, USA)).

## Results

The majority of children in the experimental group and control group were females (63.3%) while the remaining samples were males (36.7%). All the caregivers were mothers. Further analysis by performing the Chi-square analysis revealed no statistical significant differences regarding demographic characteristics of participants between the experimental and control groups showing that the two groups were properly matched (Table 1).

Mean (±SD) age of children in experimental group was 16.6 (± 8.4) months while mean (±SD) age of children in the control group was 18.1 (±7.1) months. Mean (±SD) ages of mothers in the experimental and control groups were 29.3 (±3.6) years and 28.4 (±3.5) years, respectively indicating similar age ranges in both groups. There was no statistical significant difference regarding age between the two groups showing that the two groups were matched on age properly (Table 2).

As shown in table 3, the results of analysis of variance (ANOVA) with repeated measuring showed that, in contrast to control group, PSAM scores increased in experimental group. 

**Table 1:** Demographics of children and their mothers in experimental and control groups

**Variable**	**Mean/Frequency**			**χ²**	**df**	**P-value**
**Gender of children**	Experimental group	Female	14 (70.0%)	2.13	1	0.14
		Male	06 (30.0%)			
	Control group	Female	12 (60.0%)			
		Male	08 (40.0%)			
**Education of mothers**	Experimental group	12 years and less	04 (20.0%)	0.60	2	0.70
		14 years	09 (45.0%)			
		16 years and more	07 (35.0%)			
	Control group	12 years and less	05 (25.0%)			
		14 years	08 (40.0%)			
		16 years and more	07 (35.0%)			
**Mother occupational status**	Experimental group	Employed	15 (75.0%)	3.32	1	0.60
		Homemaker	05 (25.0%)			
	Control group	Employed	11 (55.0%)			
		Homemaker	09 (45.0%)			

**Table 2:** Comparison of mean (±SD) age of mothers and their children between experimental and control groups

**Variable**		**n [mean (±SD)]**		**t**	**df**	**P-value**
**Age of children, month**	Experimental group		20 [16.6 (±8.4)]			
	Age range in experimental group		5-29			
	Control group		20 [18.1 (±7.1)]	-0.063	28	0.5
	Age range in control group		9-34			
**Age of mothers, year**	Experimental group		20 [29.3 (±3.6)]			
	Age range in experimental group		22-35			
	Control group		20 [28.4 (±3.5)]	0.520	28	0.6
	Age range in control group		24-38			

**Table 3:** Comparison of mean (±SD) scores of Parenting Self-Agency Measure (PSAM) between experimental and control groups at different stages of the study

**Follow-up**	**Post-test**	**Pre-test**	**Group**
**55 (±8.1)**	55 (±7.8)	43.7 (±8.9)	Experimental group
**52 (±7.1)**	52 (±5.3)	53.1 (±9.5)	Control group

**Table 4:** Test of inter group effects for the score of parental self efficacy

**Power of study**	**P-value**	**F**	**Mean squares**	**df**	**Sum of squares**	**Source of changes**
**Time**	407.471	2	203.731	5.612	0.006	0.83
**Group**	445.120	2	021.180	0.156	0.680	0.66
**Interactive effect of time and group**	441.121	2	232.561	5.140	0.040	0.81

As shown in table 4, the difference between the experimental and the control group showed a statistical significant main effect (F = 5.612; p < 0.006) and a significant interactive effect (F = 5.140, p < 0.04) indicated that this program significantly increased parental self-efficacy in the experimental group compared with the control group.

## Discussion

Parental self-efficacy is an important factor in increasing positive interaction between child and mother which in turn contributes to promoting mental health in children. The currents pilot study aimed to examine the effectiveness of the World Health Organization mother-child interaction program in increasing parental self-efficacy in a group of mothers of children aged 3 years or younger. Best to authors’ knowledge, this preliminary study is the first study in Iran which has taken parental self-efficacy as an important item in positive mother-child interaction in to specific consideration. 

In this preliminary study, we found that the World Health Organization international program of child-mother interaction significantly increased parental self-efficacy among the parents in this study. This study finding supports the study findings of Hanna and colleagues which showed that training sessions with the aim of increasing parental self-efficacy played an important role in increasing self-confidence among parents and promoting the abilities of parents to make more appropriate relationships with their children as an important factor which contributes to mental health (25). 

The development of group-based parenting programs has taken place in many countries over the past decade (26). Parenting programs are now being offered in many settings, and a recent review of randomized controlled trials indicated that they are effective in improving behaviour problems in 3-10 years old children (27), and in improving maternal psychosocial health in the short term, including reducing anxiety and depression and improving self-confidence (8). 

This finding in our study might be partly due to increasing self-confidence in mothers participated in this study. Increased self-confidence among them was a facilitating factor in their efficacy for a healthier interaction with their children. Coleman and Karraker in their study showed that treatment interventions designed to promote parental self-efficacy led to provide opportunities for positive parent-child interaction (20). 

Implementing this program resulted in creating an atmosphere for mothers in the study to participate in a group-based program that showed them how to increase their self-efficacy in mothering and found an opportunity to increase their parental self-efficacy with comparing their maternal behaviors with others. This issue was likely to give them feeling of well-being which increased their parental self-efficacy. This study finding is consistent with findings of Kuhn and Carter who showed that parenting self-efficacy has been associated with well-being and positive parenting outcomes. In their study, they investigated associations between maternal self-efficacy and parenting cognitions among a group of 170 mothers of children with autism. Self-efficacy appeared to be associated with well-being, agency, and feelings of guilt among these mothers (8). 

Several limitations need to be considered when interpreting the study findings. First, the present study was an introductory study and was limited to one center and with limited phases of follow-up. Indeed, we had only one follow-up phase because of limited cooperation of mothers with us. Furthermore, it was subject to the limitations of self-reports but participation in the study was voluntary, and respondents were assured that their responses would remain anonymous. 

Our study sample size was small so the results may not be generalizable to other groups of Iranian children in other parts of Iran which is subject to further studies.

There is a need for further rigorous studies of the World Health Organization international program of child-mother interaction. Larger numbers of participants should be included to increase the external validity of the research, and the measurement of a wider range of outcomes should be undertaken, including an assessment of mental health. Such studies would provide the basis for further long-term follow-up through childhood. There is conclusive evidence to show that the quality of the parent-child relationship during childhood is important for the future mental health of the child and adult. Parenting programs can improve the emotional and behavioral adjustment of children, and there is an urgent need for research to evaluate their effectiveness in preventing such problems. The preliminary evidence that has been provided in this study is a start for large-scale trials of the effectiveness of parenting programs in the primary prevention of mental health problems. 

## Authors' contributions

ZA designed the study. KT participated in conducting the study. MAM was the scientific counselor of carrying out the study procedure. LP evaluated the efficacy and accuracy of the collected clinical data. NM participated in writing the article in English. All authors read and approved the final manuscript.
